# Discovery of the possible mechanisms in kouyanqing granule for treatment of oral ulcers based on network pharmacology

**DOI:** 10.1186/s12906-020-03043-x

**Published:** 2020-08-18

**Authors:** Pan Chen, Hongliang Yao, Qing Yuan, Panlin Li, Xinning Wang, Weiwei Su, Yonggang Wang, Peibo Li

**Affiliations:** 1 Guangdong Engineering and Technology Research Center for Quality and Efficacy Re-evaluation of Post-marketed TCM, State Key Laboratory of Biocontrol and Guangdong Provincial Key Laboratory of Plant Resources, School of Life Sciences, Sun Yat-sen University, Guangzhou, 510275 China; 2 Guangdong Key Laboratory of Animal Conservation and Resource Utilization, Guangdong Public Laboratory of Wild Animal Conservation and Utilization, Drug Synthesis and Evaluation Center, Guangdong Institute of Applied Biological Resources, Guangzhou, 510260 China; 3 The school of Basic Medical Sciences, Southwest medical university, Luzhou, 646000 China

**Keywords:** Oral ulcers, Kouyanqing granule, Network pharmacology, Silico validation, Multi-component formula, Traditional Chinese medicine

## Abstract

**Background:**

Oral ulcer diseases are complex inflammatory diseases caused by multi-factors, which severely impact patient quality of life. Kouyanqing Granule (KYQG) has been used to treat inflammatory diseases of the mouth and throat, including recurrent aphthous stomatitis (RAS), traumatic ulcers, oral leukoplakia and so on. However, the underlying molecular mechanisms of KYQG in treating these diseases are still unclear. We aimed to explore the possible mechanisms in KYQG for the treatment of oral ulcers.

**Methods:**

An innovative network pharmacology method was established by incorporating targets searching and fishing, network analysis, and silico validation to discover the pharmacological mechanisms of action of KYQG for the treatment of oral ulcers. Then, we verified the reliability of this method by an animal experiment.

**Results:**

Our data indicated that a total of 47 key targets were screened, which mainly involved in three function modules including the inhibition of inflammation, the regulation of immunological response, and the suppression of oxidative stress. The implementation of these functions relies on the complex multi-pathways network, especially TNF signaling pathway and HIF-1 signaling pathway. The results of the experimental verification indicated that KYQG significantly inhibited the serum levels of cyclooxygenase-2 (COX2), matrix metalloproteinase 9 (MMP9) and tumor necrosis factor-alpha (TNF-α) in rats with oral ulcer.

**Conclusion:**

KYQG exhibited the therapeutic effects on oral ulcers probably by inhibiting inflammation, regulating immunological response, and suppressing oxidative stress through a complex multi-pathways network. Particularly, TNF signaling pathway and HIF-1 signaling pathway may play crucial roles in the protection of KYQG against oral ulcers. This work not only offers a method for understanding the functional mechanisms of KYQG for treating oral ulcer diseases from a multi-scale perspective but also may provide an efficient way for research and development of complex composition formula.

## Background

Oral ulcers are secondary lesions characterized by loss of mucosal tissue in the oral cavity. Usually, oral ulcers include conditions such as local trauma, recurrent aphthous stomatitis (RAS), viral and bacterial infections, allergic reactions, adverse drug reactions, or systemic disease [1, 2]. Oral ulcers are common. For example, RAS is the most common ulcerative disease, with a prevalence of between 5 and 60% in the population [3]. Furthermore, oral ulcers are usually expose the nerve endings of the underlying lamina propria, causing pain or soreness, particularly when eating acidic or spicy foods [4].

Kouyanqing Granule (KYQG) is made from the following five herbs: the root of *Ophiopogon japonicus* (Thunb.) Ker-Gawl., root of *Asparagus cochinchinensis* (Lour.) Merr., flower bud of *Lonicera macranthoides* Hand.-Mazz., root of *Scrophularia ningpoensis* Hemsl., and root of *Glycyrrhiza uralensis* Fisch.. KYQG is recorded in the Chinese Pharmacopoeia 2015 edition to treat mouth and throat inflammatory diseases, such as RAS, oral leukoplakia, and oral lichen planus [5]. We have previously confirmed that KYQG showed anti-inflammatory effects in an in vitro study [6]. Miao et al. reported that KYQG possesses anti-inflammatory activity and promotes the healing of phenol-induced oral ulcers in rats [7]. However, the active mechanisms by which KYQG contributing to therapeutic effects on oral ulcers remain poorly understood due to the large number of ingredients in this formulation.

**Fig. 1:**
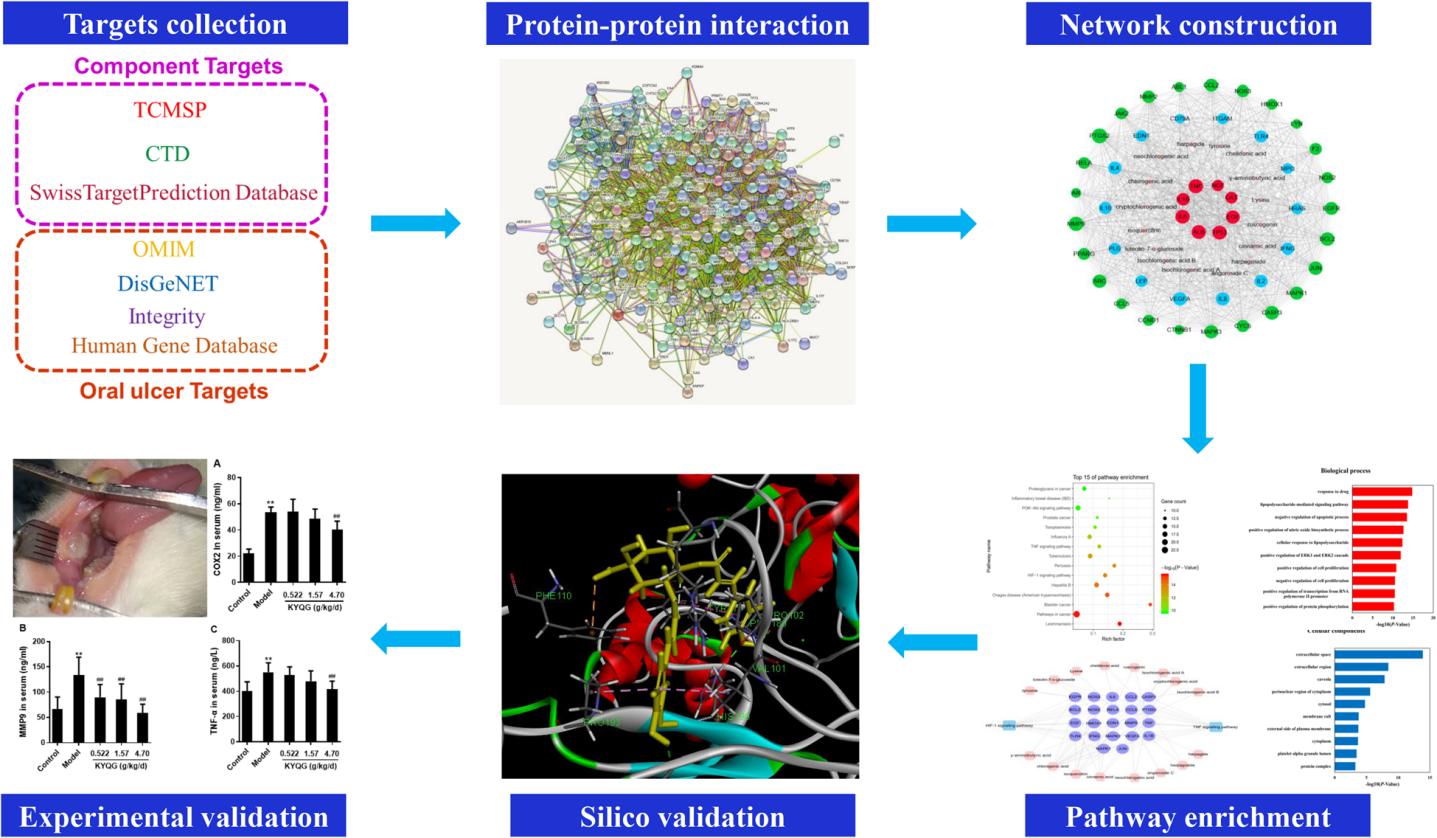
Workflow for network pharmacology approach

In the present study, an innovative network platform was established to discover the potential pharmacological mechanisms of KYQG acting on oral ulcer through the following steps as shown in Fig. 1. First, multiple targets of both active components and oral ulcer diseases were searched and fished by a comprehensive method. Second, a network pharmacology study of KYQG was established based on active component targets and oral ulcer disease targets; the crucial targets were captured through the network analysis, and the biological pathways were also enriched. Third, to validate the results of network analysis and demonstrate how the active components work on the targets, molecular docking was performed by Discovery studio 2016. Finally, an animal experiment was used to confirm the reliability of this method. In the previous studies, a rat model of oral ulcer induced by phenol was used to evaluate the effects of anti-oral ulcers [7, 8]. In our previous work, we found that sleep deprivation could aggravate oral ulcers induced by phenol damage in rats and delay the healing process [9]. In China, KYQG is specifically used to treat oral inflammatory diseases aggravated by sleep loss. Thus, we used a phenol-induced oral ulcer model with subjected sleep deprivation to validate the active mechanisms of KYQG acting on oral ulcers. The present study also provides a novel method to explain the pharmacological mechanisms of multi-component formula.

## Methods

### Collection of potential targets for the components of KYQG

**Fig. 2:**
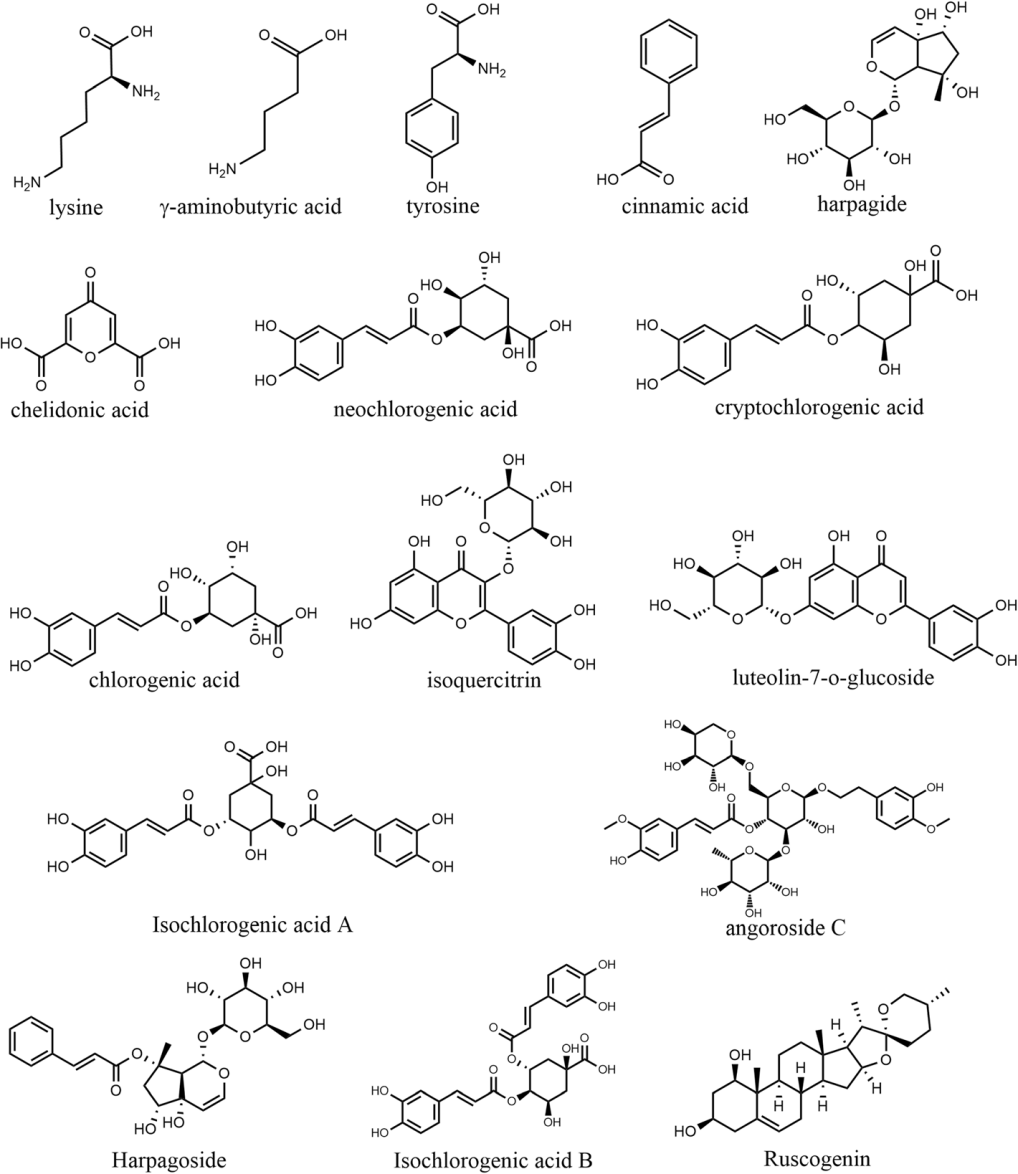
Chemical structures of the active ingredients of KYQG

In our previous study, the ingredients of KYQG were identified by using high resolution mass spectrometry technology, and we found that sixteen compounds play an important role in KYQG’s anti-inflammatory effects [6]. Therefore, the sixteen compounds were treated as the active components of KYQG, and the chemical structures of the sixteen compounds are shown in Fig. 2. At present, the potential gene targets of the active components were predicted using a comprehensive approach. To collect potential targets of sixteen active components of KYQG as many as possible, the following three databases were used: Traditional Chinese Medicine Systems Pharmacology Database and Analysis Platform (TCMSP, http://tcmspw.com/tcmsp.php) [10], Toxicogenomics Database (CTD, http://ctdbase.org/) [11], and SwissTargetPrediction Database (STPD, http://www.swisstargetprediction.ch/) [12]. To identify the component-target networks, each chemical name of the component was inputted to the “search box” on TCMSP and CTD websites respectively. STPD can estimate the most probable macromolecular targets of a small molecule based on chemical structure similarity with a library of known targets from three different species [12]. We drew each molecule of active compounds on the STPD website to predict the protein targets.

### Collection of potential targets in oral ulcers

To collect potential targets in oral ulcer, “oral ulcer/mouth ulcer” as the keywords were searched on the following resources: Online Mendelian Inheritance in Man (OMIM, https://omim.org/), a database of gene-disease associations (DisGeNET, http://www.disgenet.org/), Integrity (https://integrity.thomson-pharma.com/integrity/xmlxsl/), and Human Gene Database (Genecards, https://www.malacards.org/).

### Network construction and analysis

Protein-protein interaction (PPI) data were constructed by inputting official gene symbol to the “Multiple Proteins” search on String website [13], with organism species limited to “*Homo sapiens*” and a confidence score > 0.4. The interaction results were exported as (.tsv) file for further network analysis by using Cytoscape software. The component target-oral ulcer target (CT-OT) network was constructed based on their PPI data and visualized by Cytoscape 3.7.0 software. The component targets were mapped to the oral ulcer targets for obtaining the common targets of both. Then the common targets and their directly connected neighbors were extracted from the CT-OT network to conduct a component-key target network. In the component-key target network, nodes represent components and targets, and edges represent the interactions between nodes. The targets without interaction were removed from the network. Afterward, the topological parameters of each node in the net were analyzed by using Network Analyzer tool in Cytoscape. The topological parameters including degree, betweenness centrality, and closeness centrality were used to evaluate the significance of the nodes in networks. The nodes with “Degree” values 2-fold greater than all the network nodes’ median value, “Closeness centrality” and “Betweenness centrality” values above all the network nodes’ median value were identified as the key targets [14, 15].

### Pathway enrichment analysis for key targets

The DAVID database (https://david.ncifcrf.gov/) [16] was used to perform Gene Ontology (GO) enrichment and Kyoto Encyclopedia of Genes and Genomes (KEGG) pathway analysis, and the threshold *P* < 0.05 was set. The specific operation steps were as following: inputting the official gene symbols of the key targets and setting the species to “*Homo sapiens*,” then selecting the functional annotation tool to perform GO enrichment and KEGG analysis. The top 15 pathways were mapped to the oral ulcer disease related pathways in the CTD database to get common pathways.

### In silico validation of selected pathways

The 3D structures of the sixteen bioactive compounds were downloaded from PubChem website and saved as (.sdf) file. The structures were optimized with Discovery studio 2016. The molecular docking performance was operated by site-features directed docking (LibDock) for the study of the interactions between receptor and ligand according to previously published studies [17, 18]. In the docking study, the docking preference parameter was set to high quality, and the conformation method was set to the best. The original ligand of each protein was served as references and docked into their corresponding protein receptors in the same way described above. To evaluate the intensity of all the interactions generated, the LibDockScore was used to evaluate the receptor-ligand interactions, and the LibDockScore ratio between active compounds and original ligand was calculated.

### Animals and drug treatment

Male Sprague-Dawley rats (Eight-week-old) were purchased from Guangdong Medical Laboratory Animal Center, and housed under a 12/12-h light-dark cycle with free supply of water and food. The rats were randomly divided into five groups of seven rats each: Control group, Model group and other three groups with different doses of KYQG treated. KYQG groups were administered orally at doses of 0.522, 1.57, and 4.70 g/kg/d, respectively, from day 1 to day 9, and other groups treated with distilled water. On day 4, buccal mucosa of rats in the Model and KYQG groups were chemically damaged with phenol to create oral ulcers as described in our previous study [9]. An almost uniform round ulcer forms in the oral mucosal area on day 6. Then, the rats in the Model group and KYQG groups were subjected to sleep deprivation for 72 h as we previously described [9]. After the end of sleep deprivation, all anesthetized rats’ blood was collected from via the abdominal aortic, and then sacrificed. Serum was obtained by centrifuging the blood at 5000 rpm for 20 min at 4 °C, followed by storage at − 80 °C. The serum levels of cyclooxygenase-2 (COX2), matrix metalloproteinase 9 (MMP9), and tumor necrosis factor-alpha (TNF-α) were detected using commercial Enzyme-linked immunosorbent assay (ELISA) kits (all from Nanjing Jiancheng Bioengineering Institute, Nanjing, Jiangsu, China) according to the manufacturer’s instructions. The time-line diagram of animal experiment is depicted in Supplementary Figure 1. The animal experiments were approved by the Institutional Animal Care and Use Committee, Sun Yat-sen University (Approval No. SYSU-IACUC-2019-000181) and conducted in accordance with the institutional guidelines. Adequate measures were adopted to minimize the suffering of experimental animals.

### Method of sacrifice

After the end of sleep deprivation, the rats were anesthetized under isoflurane (Hebei Yipin Pharmaceutical Co., Ltd., Shijiazhuang, Hebei, China) delivered through airstream at a flow rate of 500 μL/min using Matrx anesthesia machine (Midmark Corp.). Then, blood was collected from all anesthetized rats via the abdominal aortic, which sacrificed the rats due to bleed to death.

### Statistical analysis

The data represent the mean values ± standard deviation (SD) for each group. The graphics presented and statistical analyses were performed using GraphPad Prism 6 program (Version 6.01). Statistical analysis was performed using the one-way ANOVA followed by the Tukey test for multiple comparisons. Results were considered significant at *P* < 0.05.

## Results

### Compound target-oral ulcers target network construction and analysis

**Fig. 3:**
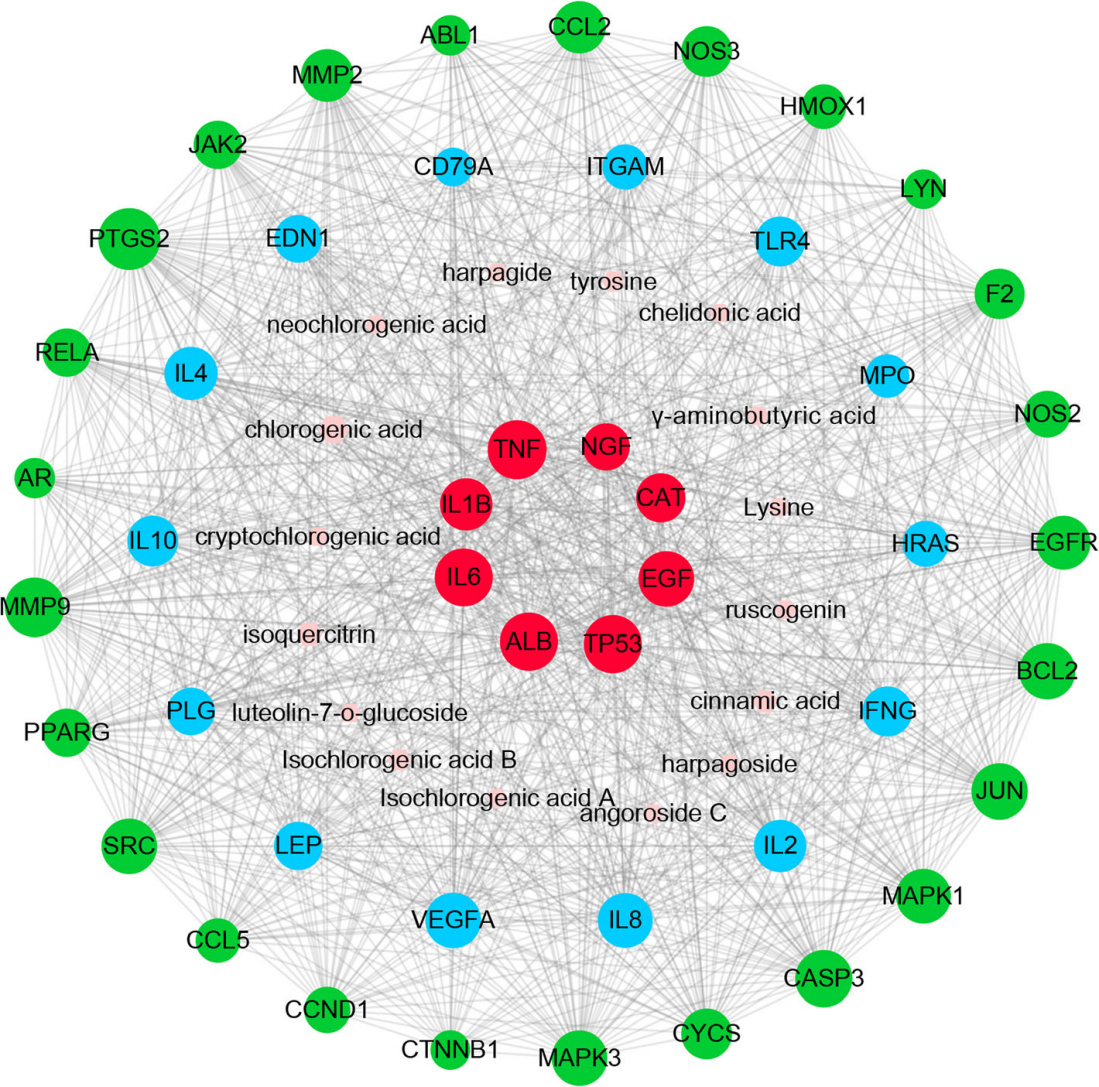
The component-key target network. Green circles, blue circles, and red circles represent the targets of compounds, oral ulcers, and the common targets for both compounds and disease, respectively. Pink hexagons represent the sixteen active compounds of KYQG. The size of circles and hexagons represent nodes degree value

A total of 274 potential targets were obtained for sixteen active components. Detailed information is described in Supplementary Table 1. By integrating data from disease databases, 97 oral ulcer-related targets were acquired (Supplementary Table 2). The CT-OT network includes 326 nodes and 4103 edges (Supplementary Figure 2). Targets of compounds were mapped to the oral ulcer-related targets to obtain 12 common targets. Then a hub PPI network (Supplementary Figure 3), including 12 common targets and their first neighbors, was extracted from the CT-OT network. The hub PPI network comprises 223 nodes and 3389 edges. A total of 47 nodes with higher value (“Degree” > 46, “Betweenness centrality” > 0.0021 and “Closeness centrality” > 0.4395) were screened as the key targets for KYQG. Then, the component-key target network was constructed by using Cytoscape software as shown in Fig. 3.

**Fig. 4:**
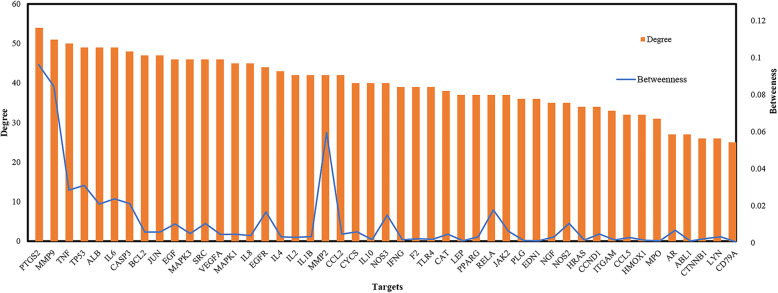
The degree and betweenness of the key targets

In the present study, to estimate the significance of the nodes in a network, degree and betweenness centrality were calculated. Degree represents the number of edges connected to the node. Betweenness centrality reflects the times of a node served as a bridge providing the shortest path between two other nodes [19]. Therefore, some targets with higher degree value and/or higher betweenness centrality value are important for the network. As shown in Fig. 4, the tendency of degree and betweenness centrality of key targets is highly correlated. The peaks of the betweenness centrality line represent the nodes that possess high betweenness centrality values despite relatively low degree values. These high betweenness centrality targets connect certain influential targets, such as MMP2, RELA, NOS3, and NOS2, which are also important. The top ten degree-ranked targets in the net are PTGS2, MMP9, TNF, TP53, ALB, IL6, CASP3, BCL2, JUN, and EGF, indicating their essential roles for the network. By further observation of this network, we found that many targets are hit by different numbers of compounds and other targets, implying the multi-targets characteristics of KYQG.

### Pathway enrichment analysis for key targets

**Fig. 5:**
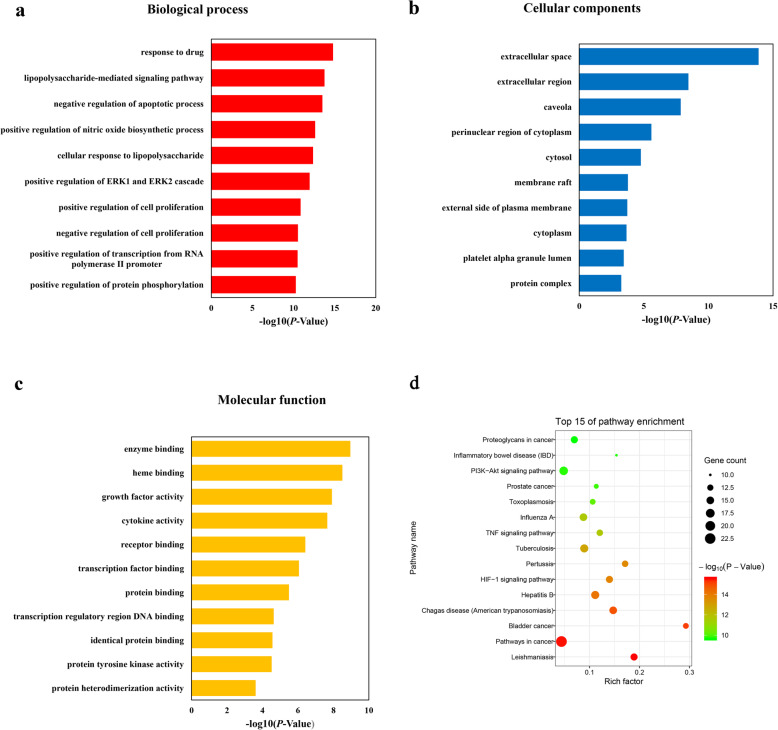
Gene Ontology (GO) and Pathway enrichment analysis of the key targets. GO terms analysis of the 47 key targets containing 3 aspects including (**a**) cellular components, (**b**) molecular function, and (**c**) biological process. (**d**) Pathway enrichment analysis of the 47 the key targets

**Fig. 6:**
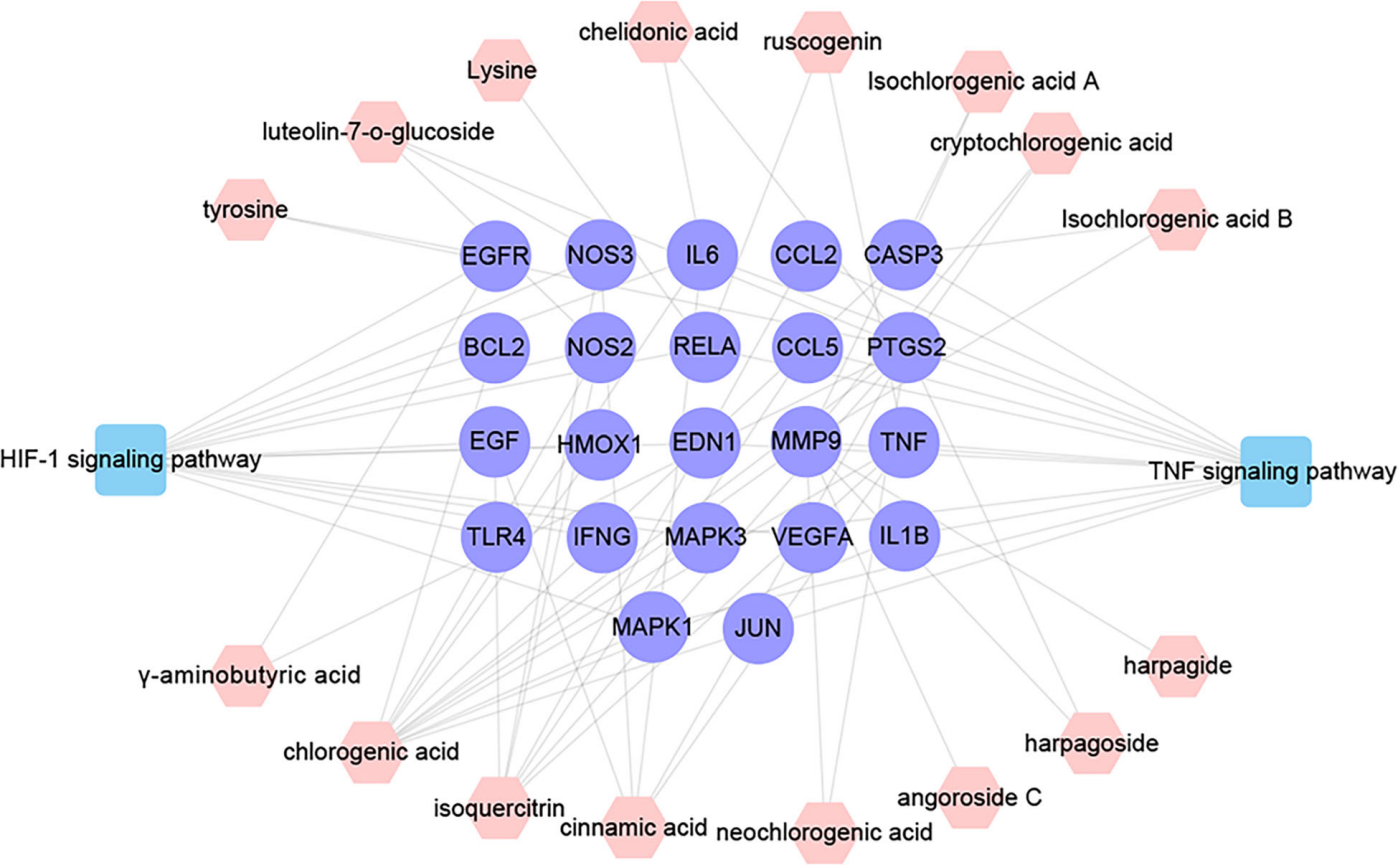
The Components-Targets-pathway network. Purple circles represent the key targets in disease pathway. Pink hexagons represent the sixteen active compounds of KYQG. Blue square represents the disease pathway

The DAVID database was used for GO analysis and KEGG pathway enrichment of 47 key targets. The results of GO analysis (Supplementary Table 3) show that 368 of 445 biological processes, 27 of 35 cell components, and 41 of 56 molecular functions enriched for these targets were recognized as *P* < 0.05. The top 10 significantly enriched terms in the biological process, cell component, and molecular function categories are shown in Fig. 5. According to the results of pathway enrichment, 101 target-related pathways have been found in the KEGG database with the adjusted *P*-value < 0.05 (Supplementary Table 4). The top 15 pathway enrichments are shown in Fig. 5d. Among these pathways, HIF-1 signaling pathway and TNF signaling pathway were related to oral ulcer diseases according to the CTD database. Then a compound-target-pathway subnetwork was built based on the two diseased related pathways, as shown in Fig. 6.

It is well known that a typical symptom of oral ulcers is the inflammation of the oral cavity. Therefore, the mechanisms of KYQG’s anti-inflammatory function were investigated. Among the top 15 pathways, several pathways are associated with inflammation, and the TNF signaling pathway is the most distinct one. Presently, 13 targets are involved in TNF-1 signaling pathway including RELA, EDN1, IL6, MAPK1, MAPK3, CCL2, CCL5, JUN, CASP3, IL1B, MMP9, PTGS2, and TNF. It has been reported that excessive production of TNF-α, IL-6, and IL-1β was associated with an increased risk of RAS development [20–22]. It has also been reported that matrix metalloproteinases have a role in mucosal pathology of the oral cavity [23–25]. In summary, the anti-inflammatory effect is an important factor for KYQG contributing to the treatment of oral ulcers.

Presently, 14 targets including BCL2, EGFR, EGF, HMOX1, IFNG, NOS2, NOS3, TLR4, VEGFA, RELA, EDN1, IL6, MAPK1, and MAPK3 are involved in HIF-1 signaling pathway which is an important regulator of the immune system. NOS2 and NOS3 are two isoforms of NOS, which produce high-level sustained NO synthesis and strongly affect adaptive immune responses [26]. NO regulates the responses of many immune and inflammatory cell types like macrophages which are associated with some of the most important immune pathologies [27]. Increased serum NO level is thought to take part in the pathogenesis of RAS [28]. Thus, NOS2 and NOS3 play an important role in the HIF-1 signaling pathway for the regulation of anti-inflammation and immune protection functions of KYQG. MAPK1 and MAPK3, two isoforms of mitogen-activated protein kinase, are of great importance in MAPK cascade and involved in the regulation of diverse biological functions. Immune response is one of several key functions regulated by MAPKs, with the release of inflammatory cytokines [29]. Actually, the immunologic disturbances play a crucial role in the etiopathogenesis of oral ulcers. Hence, it is worthwhile to explore the impact of KYQG on immune function. According to the pathway enriched results, many target genes are enriched in the pathways related to immune inflammatory diseases. For example, ten targets are enriched in T cell receptor signaling pathway (*P*-value =1.2 × 10^− 8^) and Toll-like receptor signaling pathway (*P*-value =1.9 × 10^− 8^). Eight targets are enriched in NF-kappa B signaling pathway (*P*-value =1.2 × 10^− 6^) and Jak-STAT signaling pathway (*P*-value =13.7 × 10^− 5^), and seven targets enriched in B cell receptor signaling pathway (*P*-value =4.9 × 10^− 6^). The T cell receptor signaling pathway has also been implicated in the stimulation of an abnormal Th1 immune response in RAS patients [30]. Therefore, it can be presumed that one pharmacological function of KYQG is related to the regulation of immune response for oral ulcers treatment.

Among the top 15 pathways, the PI3K-Akt signaling pathway is considered to regulate fundamental cellular functions such as cell growth, proliferation, and cell cycle [31]. It has been shown to play an important role in the inhibition of oxidative stress induced-apoptosis [32]. Furthermore, the HIF-1 signaling pathway is involved in the response to low oxygen level and modulated by TNF-α, ROS, and nitric oxide and/or NO-derived species [33]. Thus, the antioxidative effect of KYQG should also account for oral ulcer treatment.

### Computational validation of selected pathways

**Fig. 7:**
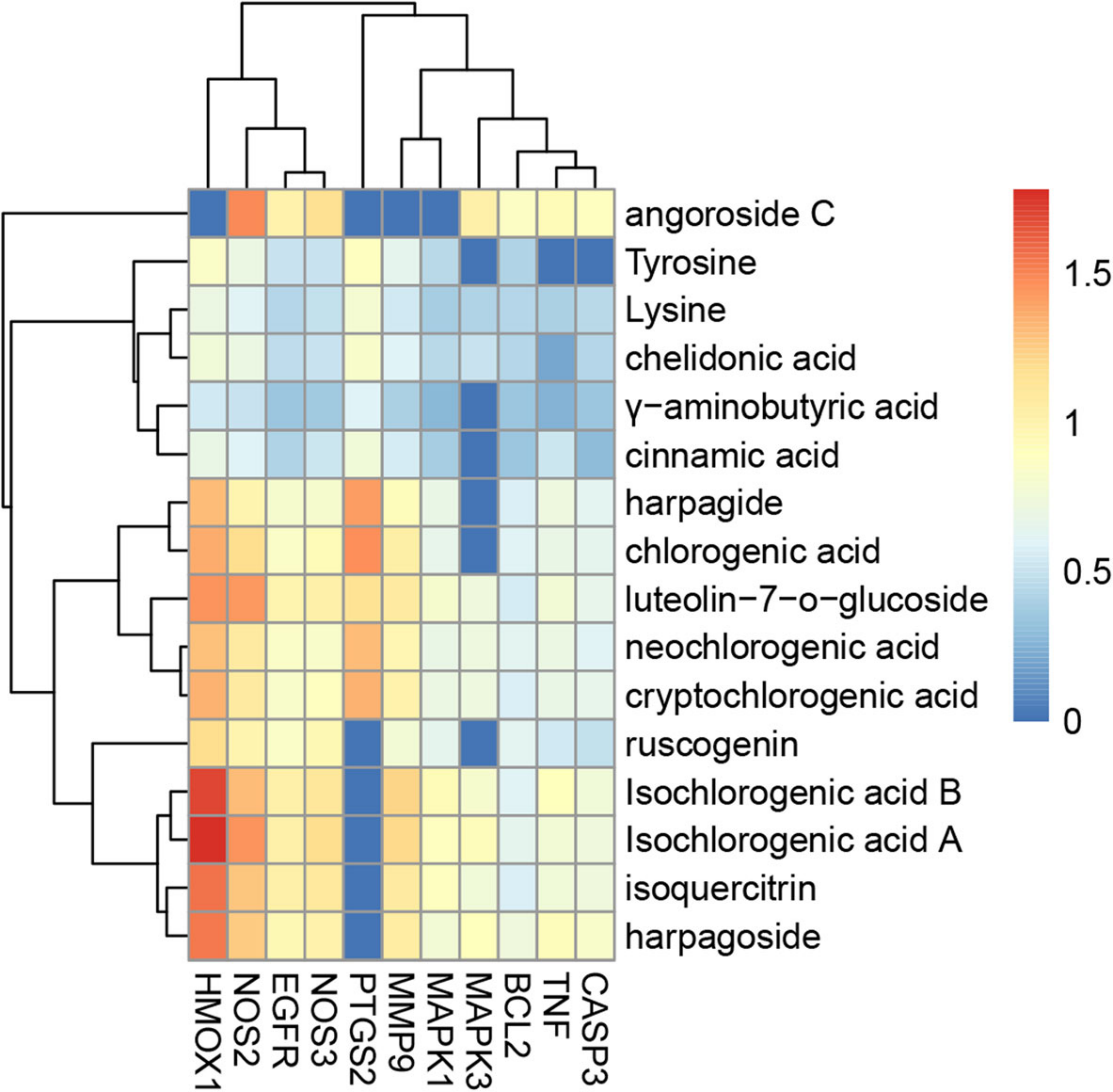
Heat map of interaction between the active components and each protein target. Estimates of interaction levels using the ratio of LibDockScores between active compounds and original ligand, colored with a gradient from blue (weak interactions) to red (strong interactions)

The targets with the protein original ligand inhibitor in both HIF-1 signaling pathway and TNF signaling pathway were selected to perform molecular docking. Herein, the highest LibDockScore value was recorded, which represents molecular docking with the best combining conformation (Supplementary Table 5). However, for some components, no proper docking results could be generated. To further describe the contributions of the components from KYQG, a heat map was presented (Fig. 7). From the docking results, we could find that different components exhibited diversity towards the target proteins selected. Isochlorogenic acid B and isochlorogenic acid A, isoquercitrin, and harpagoside showed strong interactions with HMOX1, NOS2, EGFR, and NOS3; moderate interactions with MMP9, MAPK3, MAPK1, CASP3, TNF, and BCL2; and no docking results to PTGS2. Harpagide, chlorogenic acid, luteolin-7-O-glucoside, neochlorogenic acid, cryptochlorogenic acid, and ioquercitrin showed moderated interactions with HMOX1, NOS2, EGFR, NOS3, PTGS2, and MMP9. The tyrosine, lysine, chelidonic acid, γ-aminobutyric acid, and cinnamic acid showed weak or no interactions with almost all targets. According to the docking results, the angoroside C is highly specific for NOS2, MAPK3, CASP3, TNF, and BCL2. The targets including BCL2, EGFR, HMOX1, MAPK1, MAPK3, NOS2, and NOS3 in HIF-1 signaling pathway, and PTGS2, CASP3, TNF, MMP9, MAPK1, and MAPK3 in TNF signaling pathway showed strong interactions with at least one active compounds.

**Fig. 8:**
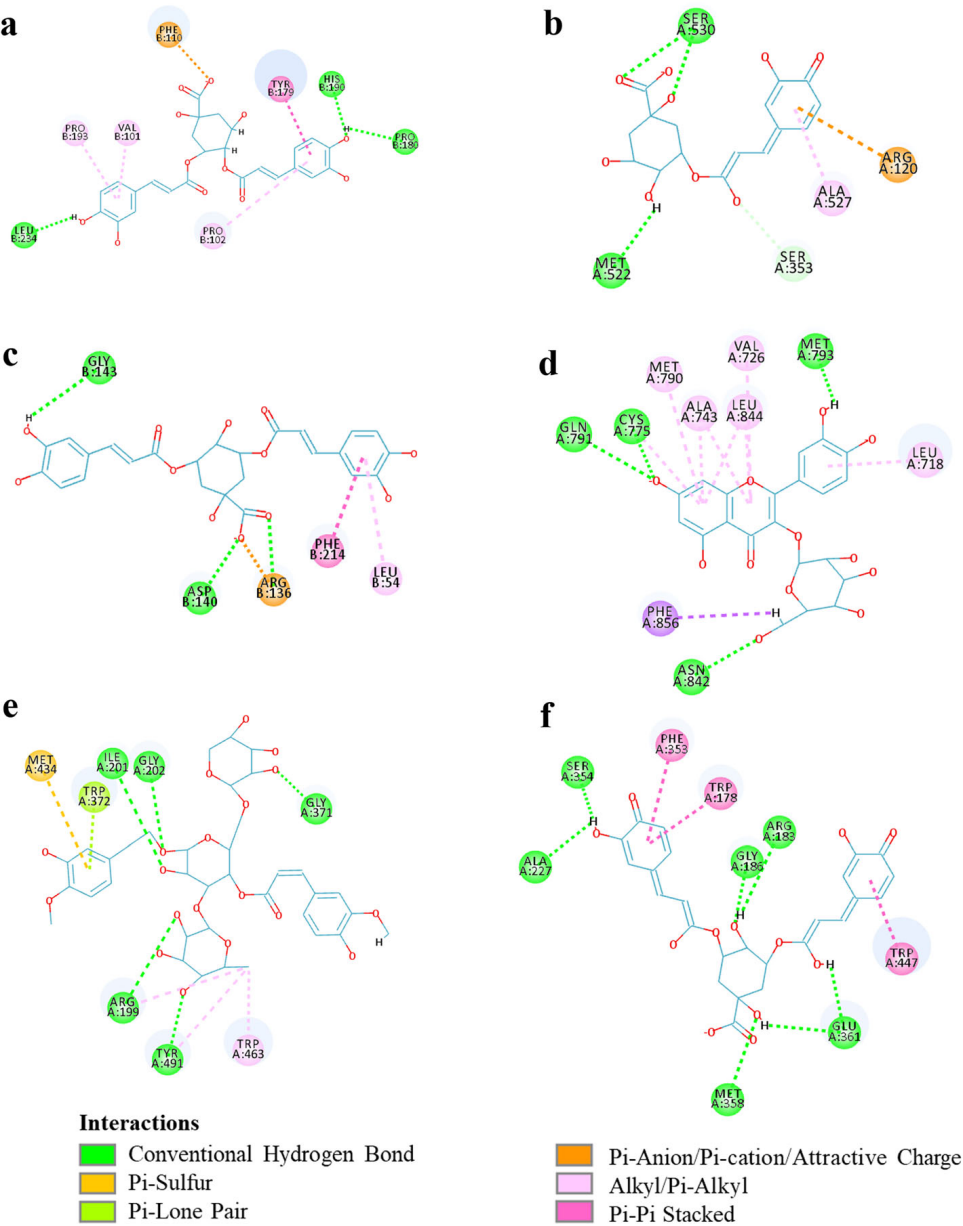
Ligand-receptor interactions on a 2D diagram. **a** Isochlorogeic acid B - MMP9, **b** Cryptochlorogenic acid-PTGS2, **c** Isochlorogenic acid A-HMOX1, **d** Isoquercitrin-EGFR, **e** Angoroside C-NOS2, **f** Isochlorogenic acid A-NOS3

A brief analysis of the characteristic receptor-ligand interactions of the component ligand with higher LibDockScore than the other component ligands and original ligand was performed. As shown in Fig. 8a, isochlorogenic acid B forms three hydrogen bonding interactions with the TYR179 and ASP177 amino acid residues with the MMP9 receptor. Three Pi-Alkyl interactions were observed with the amino acid residues PRO193, VAL101, and PRO102. Pi-Pi stacked interaction with amino acid residue PHE110 was observed. Coincident with the published literature [34], TYR179, VAL101, and PHE110 were the active amino acid residues of MMP9. The results could describe the receptor-ligand interactions for the binding pattern and possible action mechanisms. Thus, such results and methodology were considered to be reliable.

The complex of PTGS2 with cryptochlorogenic acid is stabilized by two hydrogen bond interactions with SER530 and MET522 (Fig. 8b). The docking results are in line with previous studies that hydrogen bonds are formed between the carboxylate group of the inhibitor and the hydroxyl oxygen of SER530 [35]. Previous studies reported that SER530 is an important determinant of time-dependent inhibition of PTGS2 [35, 36]. These results further verify the anti-inflammation effects of cryptochlorogenic acid. Furthermore, other interactions, such as Pi-alkyl and Pi-Cation, contribute to the binding affinity of cryptochlorogenic acid with PTGS2.

According to Fig. 8c, isochlorogenic acid A forms three hydrogen bonding interactions with the GLY143, ASP140, and ARG136 amino acid residues of HOMX1 receptor. ARG136 amino acid residue also forms Attractive Charge interaction with the ligand. There is a Pi-Pi T-shaped interaction with PHE214 residue, and a Pi-Alkyl interaction with LEU54 residue was observed. Previous studies have shown that ASP140 is critical for both human and rat heme oxygenase-1 enzyme activity [37–39].

The highest docking score is also observed in the strong interaction of isoquercitrin with the EGFR receptor (Fig. 8d). Isoquercitrin forms four hydrogen bonding interactions with the side chain of GLN791, CYS775, MET793, and ASN842 amino acid residues; six Pi-Alkyl interactions with CYS775, MET790, ALA743, VAL726, LEU844, and LEU718 amino acid residues were identified; it also forms Pi-sigma interaction with PHE856 amino acid residue.

The angoroside C was nicely bound to the active site of NOS2 by five conventional hydrogen bonds with ILE201, GLY202, ARG199, TYR491, and GLY371 residue and one Pi-Sulfur bond with MET434 residue (Fig. 8e). Besides, other interactions, such as Pi-alkyl, alkyl, and carbon-hydrogen bonds, contribute to the binding affinity of angoroside C with NOS2.

Isochlorogenic acid A showed six hydrogen bond interactions with NOS3 receptor by ALA227, SER354, GLY186, MET358, ARG183, and GLU361 residue, as well as Pi-Pi stacked interactions by PHE353, TRP178, and TRP447 residue were observed (Fig. 8f).

### Experimental validation

**Fig. 9:**
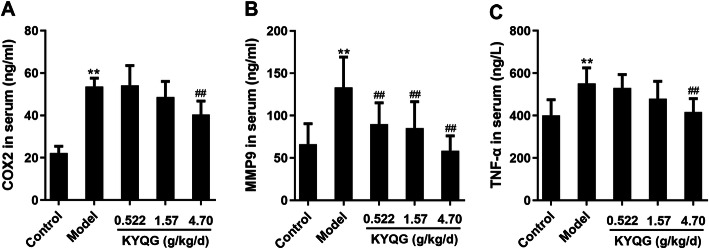
The concentrations of COX2 (**a**), MMP9 (**b**) and TNF-α (**c**) in serum. Data are expressed as mean ± SD (*n* = 7). ** *P* < 0.01 compared with Control group; ^##^ *P* < 0.01 compared with Model group

We preliminarily inferred the active mechanisms of KYQG by using present network pharmacology method, but such a method needs for further experimental verification. In order to demonstrate the reliability of our method, we conducted the animal experiment on an oral ulcer model in rats. In the component-key target network, PTGS2, referred to as cyclooxygenase 2 (COX2), MMP9, and TNF-α are the top three degree-ranked targets, which were considered as the important roles in oral ulcers treatment of KYQG. Therefore, we detected the COX2, MMP9, and TNF-α levels in serum. As shown in Fig. 9, KYQG (4.7 g/kg/d) significantly inhibited the COX2, MMP9 and TNF-α (*P* < 0.01) levels in serum. These results suggest that the therapeutic effects of KYQG were related to the decreases of COX2, MMP9, and TNF-α, which are consistent with our network pharmacology study. Thus, these combined results suggest our network pharmacology method was considered to be reliable.

## Discussion

Oral ulcers are complex inflammation diseases caused by multi-factors and impact patients’ quality of life severely. The etiology of oral ulcerations is not entirely clear, and it has been reported that several local, systemic, iatrogenic, immunologic, genetic, allergic, nutritional, and microbial factors are responsible [40]. Many studies have confirmed the crucial role of immunologic disturbances in the etiopathogenesis of RAS as well [41]. However, for oral ulcers of unknown origin or related to autoimmune diseases, there is no specific treatment for it.

Presently, a comprehensive network pharmacology method was established to study the therapeutic targets and possible mechanisms of KYQG for treating oral ulcers.

The mechanisms of KYQG for treating oral ulcers are fulfilled at least through its three function modules, i.e., the inhibition of inflammation, the regulation of immunological response, and the suppression of oxidative stress. And the implementation of these functions relies on a smooth run of the complex multi-pathways network, especially TNF signaling pathway and HIF-1 signaling pathway. Moreover, we adopted in silico validation for studying the interactions between the bioactive compounds and key targets in the predicted pathways. In silico validation of key targets for bioactive components offers an alternative path for the exploration of ligand-target interactions and action mechanisms. We could conclude that the different components display diverse interactions with these targets in the predicted pathways. The results demonstrated a synergistic effect mode of the KYQG in its oral ulcer treatment effects. For example, isochlorogenic acid B could interact with MMP-9 by active site residues TYR179, VAL101, and PHE110; cryptochlorogenic acid interacts with PTGS2 by active site residue SER530, and Isochlorogenic acid A interacts with HMOX-1 by active site residue ASP140. Consequently, the strong interactions between these active components and targets are the basis of these molecules’ biological activities. Usually, TCM formula tends to play a role in a holistic manner for the treatment of complex disease. The molecular docking study helps us to better understand the synthetic functions of multi-components in KYQG for oral ulcer treatment. To demonstrate the reliability of our method, we conducted the animal experiment on an oral ulcer model in rats and detected the serum levels of COX2, MMP9, and TNF-α. MMP9 has a significant association with RAS [23], and COX2 is closely related to oral lichen planus [42]. Furthermore, doxycycline [24] and rofecoxib [3] were used as inhibitors of MMPs and COX2, respectively, and have been used in the treatment of oral ulcers. TNF-α is a pro-inflammatory cytokine whose excessive production is associated with an increased risk of RAS development [22]. Coincident with the network analysis, KYQG showed inhibitory effects on MMP9, COX2, and TNF-α in the animal experiments. Such results demonstrate our network pharmacology method was considered to be reliable.

## Conclusion

In summary, KYQG is advantageous as complementary medicine in the multimodal treatment of oral ulcer disease due to its multi-component and multi-target characteristics. KYQG exhibited the therapeutic effects on oral ulcers probably by inhibiting inflammation, regulating immunological response, and suppressing oxidative stress based on 47 key targets, such as MMP9, COX2, and TNF-α. Particularly, TNF signaling pathway and HIF-1 signaling pathway may play crucial roles in the protection of KYQG against oral ulcers. Besides, this work is expected to be useful for carrying out a systematic study of multi-component formula as well as developing novel bioactive ingredients.

## Supplementary information

**Additional file 1: Supplementary Table 1.** The table of compound targets.

**Additional file 2: Supplementary Table 2.** The table of oral ulcer related targets.

**Additional file 3: Supplementary Table 3.** The results of GO analysis.

**Additional file 4: Supplementary Table 4.** The results of pathway enrichment.

**Additional file 5: Supplementary Table 5.** LibDockScore results of molecular docking.

**Additional file 6: Supplementary Figure 1.** The time-line diagram of animal experiment.

**Additional file 7: Supplementary Figure 2.** The component target-oral ulcer target (CT-OT) network.

**Additional file 8: Supplementary Figure 3.** The protein-protein interaction network.

## Data Availability

The data used and/or investigated during the present study are available from the corresponding author upon reasonable request.

## Abbreviations

COX2: Cyclooxygenase-2
CTD: Toxicogenomics Database
CT-OT: Component target-oral ulcer target
DAVID: Database for Annotation Visualization and Integrated Discovery
DisGeNET: a database of gene-disease associations
ELISA: Enzyme-linked immunosorbent assay
GO: Gene Ontology
KEGG: Kyoto Encyclopedia of Genes and Genomes
KYQG: Kouyanqing Granule
MMP9: Matrix metalloproteinase 9
OMIM: Online Mendelian Inheritance in Man
PPI: Protein-protein interaction
RAS: Recurrent aphthous stomatitis
STPD: SwissTargetPrediction Database
TCMSP: Traditional Chinese Medicine Systems Pharmacology Database and Analysis Platform
TCM: Traditional Chinese medicine
TNF-α: Tumor necrosis factor-alpha
