# Supplementary material for: Discovery of the possible mechanisms in kouyanqing granule for treatment of oral ulcers based on network pharmacology
Source: BMC Complement Med Ther. 2020 Aug 18;20:258. doi: 10.1186/s12906-020-03043-x (PMC7436979; doi:10.1186/s12906-020-03043-x)
Supplement: Supplementary file 5 — Additional file 5: Supplementary Table 5. LibDockScore results of molecular docking. [file 12906_2020_3043_MOESM5_ESM.docx]

Supplementary Table 5. LibDockScore results of molecular docking

| Receptors  Ligands | PTGS2 (5IKQ) | MMP9 (5UE4) | TNF (4TWT) | CASP3 (2Y0B) | BCL2 (6GL8) | MAPK3 (4QTB) | MAPK1 (4QTA) | EGFR (5UG9) | NOS3 (4D1P) | NOS2 (3E7G) | HMOX1 (3CZY) |
| --- | --- | --- | --- | --- | --- | --- | --- | --- | --- | --- | --- |
| Original ligand | 89.679 | 136.434 | 158.889 | 176.053 | 164.005 | 172.209 | 185.406 | 138.843 | 148.932 | 125.364 | 82.4053 |
| lysine | 71.9214 | 73.9786 | 64.1816 | 80.1101 | 71.946 | 72.2494 | 69.9558 | 61.3603 | 72.2698 | 77.8349 | 57.6999 |
| γ-aminobutyric acid | 53.4304 | 54.0092 | 42.3622 | 61.186 | 55.9121 | -- | 51.8142 | 47.3322 | 54.684 | 63.8638 | 44.0948 |
| chelidonic acid | 73.5823 | 82.1995 | 33.0324 | 75.7319 | 70.3447 | 86.6806 | 83.559 | 65.1657 | 74.7534 | 87.8931 | 62.1329 |
| tyrosine | 78.7262 | 88.4341 | -- | -- | 69.4219 | -- | 84.913 | 69.8199 | 75.7726 | 87.9846 | 70.1883 |
| harpagide | 127.386 | 124.105 | 114.223 | 111.088 | 95.2139 | -- | 126.961 | 112.705 | 119.765 | 124.856 | 107.652 |
| neochlorogenic acid | 118.165 | 132.581 | 111.546 | 109.623 | 104.744 | 125.549 | 125.894 | 118.264 | 124.355 | 135.339 | 106.385 |
| chlorogenic acid | 131.25 | 141.174 | 109.64 | 113.897 | 100.252 | -- | 124.326 | 117.02 | 139.204 | 148.764 | 111.646 |
| cryptochlorogenic acid | 120.294 | 137.361 | 109.011 | 118.262 | 96.2391 | 122.972 | 129.26 | 113.855 | 130.399 | 134.267 | 110.883 |
| isoquercitrin | -- | 148.263 | 119.232 | 130.658 | 95.6133 | 130.511 | 162.701 | 143.487 | 162.279 | 159.199 | 128.677 |
| luteolin-7-o-glucoside | 102.549 | 147.492 | 121.999 | 118.928 | 91.5716 | 128.829 | 150.586 | 136.511 | 153.327 | 180.191 | 119.781 |
| isochlorogenic acid B | -- | 166.048 | 143.93 | 133.608 | 101.668 | 139.705 | 172.761 | 143.42 | 164.682 | 165.286 | 140.508 |
| isochlorogenic acid A | -- | 164.469 | 122.313 | 131.627 | 107.625 | 157.241 | 163.084 | 142.893 | 176.811 | 182.733 | 146.885 |
| angoroside C | -- | -- | 147.843 | 156.872 | 141.722 | 164.34 | -- | 140.126 | 174.501 | 186.528 | -- |
| harpagoside | -- | 144.047 | 145.593 | 145.165 | 120.128 | 153.593 | 144.141 | 133.174 | 151.069 | 157.511 | 127.767 |
| cinnamic acid | 67.3545 | 76.7589 | 82.321 | 51.936 | 56.7284 | -- | 71.6629 | 58.8957 | 77.9021 | 74.6779 | 57.055 |
| ruscogenin | -- | 106.383 | 87.7903 | 87.0212 | 103.463 | -- | 120.374 | 116.916 | 142.334 | 123.966 | 97.3593 |
