# Supplementary figures and images for: Discovery of the possible mechanisms in kouyanqing granule for treatment of oral ulcers based on network pharmacology
Source: BMC Complement Med Ther. 2020 Aug 18;20:258. doi: 10.1186/s12906-020-03043-x (PMC7436979; doi:10.1186/s12906-020-03043-x)

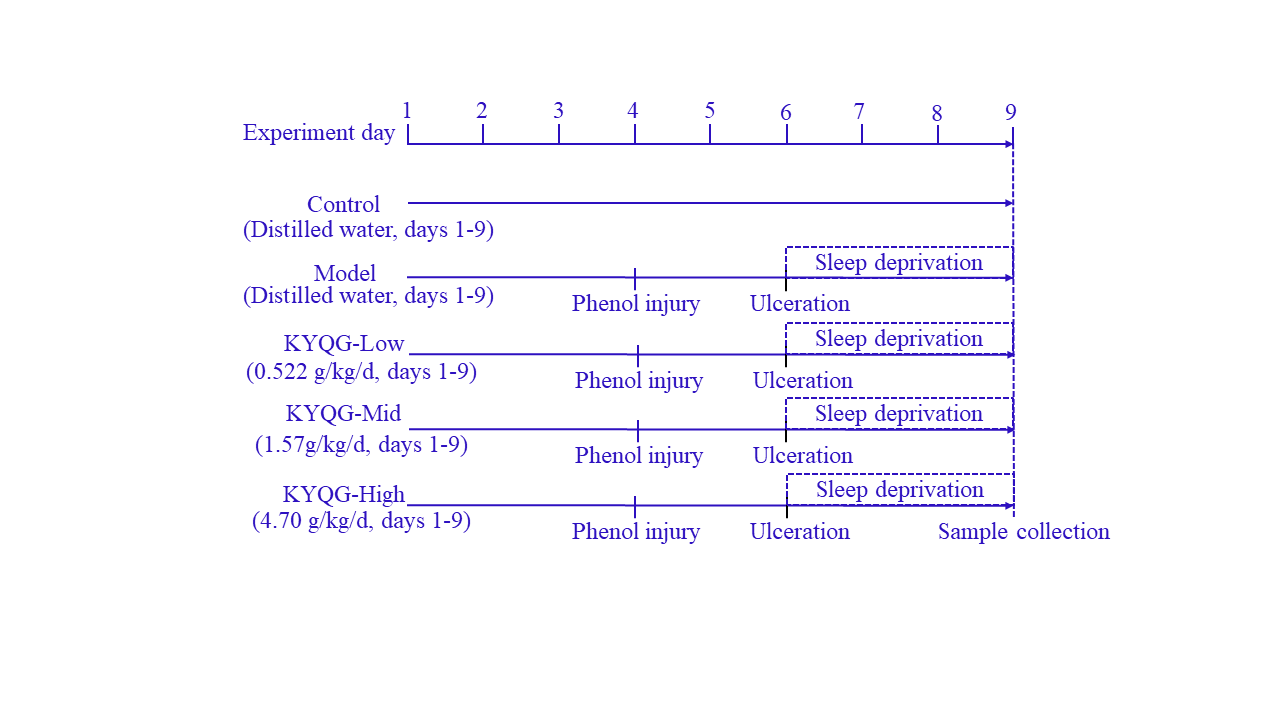

Supplement: Supplementary file 6 — Additional file 6: Supplementary Figure 1. The time-line diagram of animal experiment. [file 12906_2020_3043_MOESM6_ESM.tif]

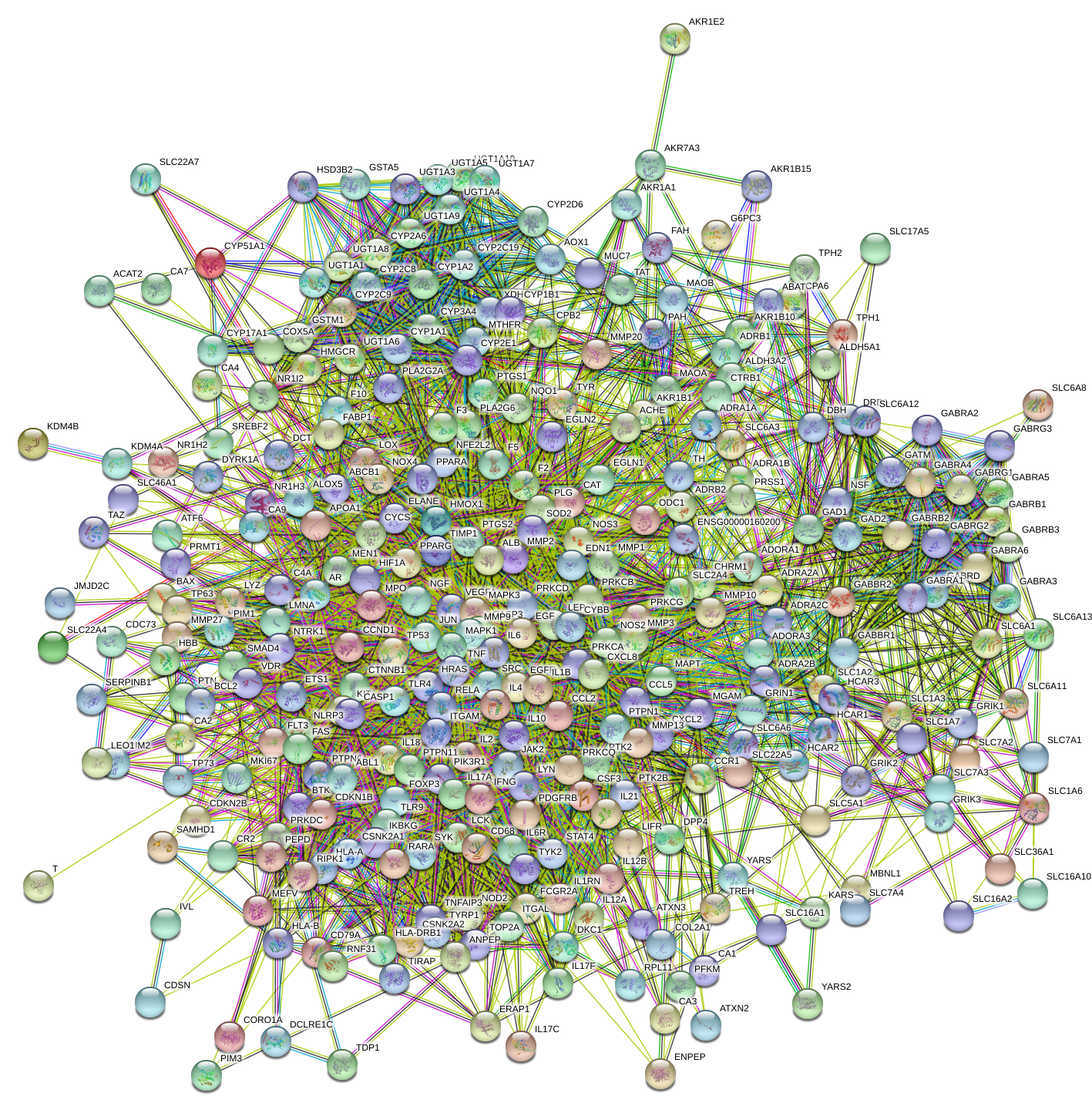

Supplement: Supplementary file 7 — Additional file 7: Supplementary Figure 2. The component target-oral ulcer target (CT-OT) network. [file 12906_2020_3043_MOESM7_ESM.tif]

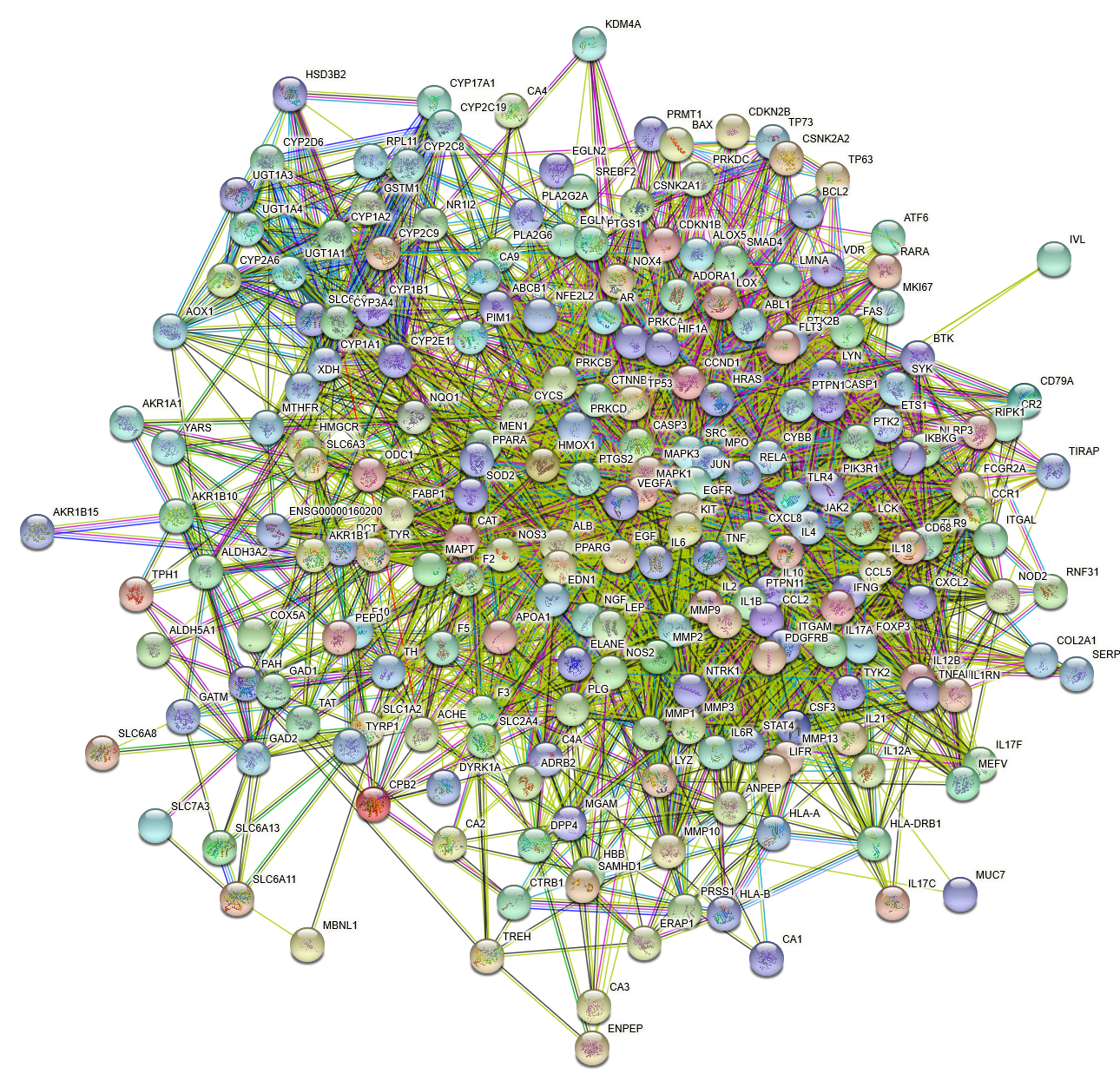

Supplement: Supplementary file 8 — Additional file 8: Supplementary Figure 3. The protein-protein interaction network. [file 12906_2020_3043_MOESM8_ESM.tif]
